# Educational Attainment Better Protects Non-Latino than Latino People Against Diabetes Mellitus

**DOI:** 10.31586/gjcd.2025.1099

**Published:** 2025-05-04

**Authors:** Shervin Assari, Hossein Zare

**Affiliations:** 1 Marginalized-Related Diminished Returns (MDRs) Research Center, Los Angeles, CA, USA; 2 Department of Internal Medicine, Charles R. Drew University of Medicine and Science, Los Angeles, CA, USA; 3 Department of Psychiatry, Charles R. Drew University of Medicine and Science, Los Angeles, CA, USA; 4 Department of Family Medicine, Charles R. Drew University of Medicine and Science, Los Angeles, CA, USA; 5 Department of Urban Public Health, Charles R. Drew University of Medicine and Science, Los Angeles, CA, USA; 6 Department of Health Policy and Management, Johns Hopkins Bloomberg School of Public Health, Baltimore, MD, USA; 7 School of Business, University of Maryland Global Campus (UMGC), Adelphi, USA

**Keywords:** Diabetes, Educational Attainment, Minorities’ Diminished Returns, Latinos, Health Disparities, Structural Inequality

## Abstract

**Background:**

High educational attainment is a well-recognized protective factor against health problems such as diabetes. However, the theory of Minorities’ Diminished Returns (MDRs) suggests that this protective effect is weaker for ethnic minorities compared to non-Latino Whites. This diminished effect is thought to result from structural inequalities, such as lower-quality education and fewer occupational opportunities, faced by ethnic minorities.

**Objective:**

This study examined the protective effect of years of schooling—used as a proxy for educational attainment—on diabetes mellitus (DM), overall and by ethnicity. Based on the MDRs framework, we hypothesized that the protective effect of education would be weaker for Latino individuals compared to non-Latinos.

**Methods:**

Data were drawn from the 2012 wave of the Understanding America Study (UAS), a nationally representative, internet-based panel. The outcome of interest was self-reported doctor diagnosis of DM. Logistic regression models were used to assess the association between educational attainment and DM, with an interaction term to explore differences between Latino and non-Latino individuals. Models were adjusted for age, sex, employment, immigration status, and marital status. Findings were presented as adjusted odds ratios (OR), p-values, and 95% confidence intervals (CIs).

**Results:**

Higher educational attainment was associated with lower odds of DM in both Latino and non-Latino individuals (p < 0.001). An interaction between education and ethnicity (p < 0.05) indicated that the protective effect of education was weaker for Latino individuals compared to non-Latinos.

**Conclusion:**

The findings align with the MDRs framework, which suggests that the health benefits of education are not equally distributed across ethnic groups. For Latino individuals, structural barriers such as lower educational quality and labor market discrimination may limit the protective effect of education against DM. While education is a key determinant of health, its unequal returns contribute to ethnic health disparities. Policymakers must address structural inequalities in education and employment that disproportionately affect ethnic minorities. Tackling these disparities through multi-sector policy interventions will require bipartisan political support.

## 1. Background

Educational attainment is widely regarded as a key social determinant of health [1–5]. Individuals with more years of schooling and higher educational achievement tend to have better health outcomes, including lower rates of chronic diseases like diabetes mellitus (DM) [6–17]. Several factors contribute to this protective effect, such as enhanced health literacy, improved nutrition, better access to healthcare resources, reduced stress, and healthier lifestyle choices, including greater physical activity [18–22]. Both the quantity and quality of education play a critical role in promoting overall health [20, 23]. Conversely, when the quality or access to education is compromised, the associated health benefits may be reduced [24–28].

A body of research has shown that each additional year of education correlates with reduced risk of DM and other chronic health conditions [18, 29, 30]. However, the assumption that education uniformly protects against health problems has been challenged by some studies, suggesting that its effects may not be consistent across different populations and social contexts [31]. This indicates that education’s influence on health outcomes, such as DM, can vary depending on the individual’s social and environmental conditions [32–36].

Although education is generally protective against many adverse health outcomes, the extent of this protection is not uniformly experienced across different ethnic and social groups [37]. The Minorities’ Diminished Returns (MDRs) theory [38, 39] suggests that the positive health effects of socioeconomic factors—such as educational attainment—are less pronounced for ethnic minorities compared to non-Latino Whites. Even when ethnic minority individuals achieve similar levels of education, they often experience fewer health benefits, such as lower DM risk, due to systemic structural inequalities. These inequities arise from disparities in education quality, job opportunities, and access to resources, reflecting broader societal inequalities that extend beyond poverty or low education alone [37, 40–46].

Structural barriers embedded in systems like education, employment, finance, and law enforcement disproportionately impact minority communities, limiting their ability to fully translate educational attainment into better health outcomes. Historical and ongoing social marginalization reduces the ability of these groups to reap the health benefits associated with higher education [37]. As a result, MDRs manifest as a reduced protective effect of education on health outcomes, including DM, among minority populations [37, 47–56].

Numerous studies have documented MDRs in various areas, including health behaviors, mental health, and physical health outcomes [57–69]. For example, while educational attainment is generally associated with increased income and wealth, Black individuals experience lower financial returns on their education compared to their White counterparts [45, 70, 71]. This disparity can be attributed to factors such as labor market discrimination, residential segregation, and unequal access to high-quality education, which can result in highly educated Black individuals having less favorable job prospects and attending lower-quality schools than their White peers [72–74].

In a similar vein, the positive effects of education on health-related behaviors (e.g., substance use) and mental health (e.g., depression [63, 75], anxiety [76], and suicide [63, 77–79]) are often weaker for Latino and Black populations than for non-Latino Whites. Studies also suggest that the health benefits of education, including lower DM prevalence [83], improved dietary habits [81, 82], reduced heart disease [83], and decreased mortality [84], are less pronounced for Black individuals compared to Whites.

Although MDRs have been widely studied across numerous outcomes, limited research has focused specifically on the effect of MDRs on DM risk, particularly among Latino populations. This gap in the literature is significant, as Latinos represent a growing demographic in the U.S., with increasing levels of educational attainment. Investigating how the health benefits of education differ between Latino and non-Latino groups is essential for addressing both educational and health disparities. While some research has explored MDRs in relation to DM and its correlates [32–36], much of this work has focused on Black-White differences, highlighting the need for further investigation in Latino populations.

## 2. Methods

The Understanding America Study (UAS) is a nationally representative, internet-based panel survey managed by the University of Southern California (USC) aimed at providing insights into various social, economic, and health-related issues across the U.S. population [39, 85–88]. Panel members are selected through probability-based sampling from post-office delivery sequence files. To ensure a diverse and representative sample, individuals without internet access are provided with internet-enabled tablets and service, allowing them to participate. At the time of data collection, the UAS panel included over 9,600 participants, with nearly 5,000 individuals aged 50 or older. The UAS collects extensive background information from participants, covering areas such as well-being, retirement, personality traits, and cognitive functioning. These core surveys, along with periodic BMI measurements, are conducted annually or biennially, providing participants with regular opportunities to engage with the study through online surveys.

For this study, data were drawn from the 2012 UAS wave, with a focus on a specific subset of participants across various age groups (30–50, 50–64, 65–74, and 75+). Participants were asked whether they had ever been diagnosed with diabetes mellitus (DM) by a doctor, with responses categorized as a binary variable (1 for yes, 0 for no).

### 2.1. Data Analysis

To explore ethnic group differences in DM prevalence, a Chi-square test was initially performed. This test evaluated the group differences in DM rates between Latinos and non-Latinos. Additionally, t-tests were used to compare the age and education levels of these ethnic groups. Following these initial comparisons, logistic regression models were utilized to examine the relationship between educational attainment (measured in years of schooling) and DM, while controlling for other variables such as ethnicity, age, sex, employment status, and marital status. Two models were analyzed: Model 1 was the baseline model that included education, ethnicity, and other relevant predictors without interaction terms. Model 2 incorporated an interaction term between ethnicity and education to assess whether the relationship between educational attainment and DM varied between Latino and non-Latino individuals. Results from the logistic regression models were presented as odds ratios (OR), p-values, and 95% confidence intervals (CIs). This method allowed for the examination of any significant differences in the protective effects of education on DM prevention between ethnic groups, with particular attention to the concept of Minorities’ Diminished Returns (MDRs) in Latino populations compared to non-Latino populations.

### 2.2. Ethics

All participants in this study were previously enrolled in the UAS panel and had given consent for their participation in UAS-related research. However, for this specific study, the USC Institutional Review Board (IRB) required an expanded consent process. This process clearly informed participants that individuals with progressive cognitive impairments, which might affect their ability to provide informed consent, were not eligible to take part. To ensure that participants fully understood their rights, they were asked to answer three multiple-choice questions about these rights before providing their final consent. This study received approval from the USC IRB.

## 3. Results

### 3.1. Descriptives

Table 1 presents descriptive statistics for the overall sample and by ethnicity (Non-Latino and Latino). The average age of participants was 48 years (SD = 16), with Non-Latino individuals being older on average (M = 50, SD = 15) compared to Latino individuals (M = 39, SD = 14). Educational attainment was slightly higher for Non-Latinos (M = 11.21 years, SD = 2.22) compared to Latinos (M = 10.34 years, SD = 2.46). In terms of race, 90.5% of the total sample identified as White, with 89.6% of Non-Latinos and 97.0% of Latinos identifying as such. The proportion of Black participants was 9.5% overall, with 10.4% among Non-Latinos and 3.0% among Latinos. Gender distribution showed that 42.5% of the overall sample were men and 57.5% were women. Among Non-Latinos, 43.8% were men and 56.2% were women, while for Latinos, 33.7% were men and 66.3% were women. Regarding marital status, 57.2% of the overall sample were married, with a slightly higher percentage of Non-Latinos being married (58.1%) compared to Latinos (50.7%). Labor market participation was similar across groups, with 58.2% of the overall sample being employed, 57.7% of Non-Latinos, and 61.2% of Latinos reporting employment. There were notable differences in nativity, as 93.6% of the overall sample were U.S.-born. However, only 73.7% of Latinos were U.S.-born compared to 96.4% of Non-Latinos. Finally, regarding diabetes mellitus (DM), 11.5% of the overall sample had been diagnosed with DM, with 11.7% of Non-Latinos and 9.8% of Latinos reporting a DM diagnosis. Significant differences were observed between Latino and Non-Latino groups for age, education, gender, marital status, and U.S. nativity (p < 0.05).

### 3.2. Regression Results without Interaction Term

Table 2 presents the results of the logistic regression analysis with diabetes mellitus (DM) as the outcome. Overall, age, education, employment status, and Black ethnicity were significant predictors of DM in this model. Overall, age, education, employment status, and Black ethnicity were significant predictors of DM in this model. Educational attainment (years of schooling) was significantly associated with lower odds of DM (OR = 0.877, 95% CI [0.848, 0.907], p < 0.001), suggesting a protective effect of education on DM risk. Employment was also protective, with individuals who were working showing lower odds of DM (OR = 0.696, 95% CI [0.585, 0.826], p < 0.001). However, no significant associations were found between DM and sex (OR = 0.922, 95% CI [0.787, 1.081], p = 0.320), Hispanic ethnicity (OR = 1.217, 95% CI [0.922, 1.607], p = 0.166), immigrant status (OR = 1.134, 95% CI [0.798, 1.610], p = 0.484), or marital status (OR = 1.066, 95% CI [0.907, 1.253], p = 0.440). Black individuals had significantly higher odds of DM compared to non-Black participants (OR = 1.719, 95% CI [1.347, 2.193], p < 0.001). Age was significantly associated with higher odds of DM (OR = 1.038, 95% CI [1.032, 1.044], p < 0.001), indicating that each additional year of age increased the odds of DM.

Table 3 shows the logistic regression results with an interaction term between ethnicity and education predicting diabetes mellitus (DM). Age remained significantly associated with higher odds of DM (OR = 1.038, 95% CI [1.032, 1.044], p < 0.001), indicating that older individuals had a higher likelihood of DM. Education continued to show a protective effect, with higher years of schooling being associated with lower odds of DM (OR = 0.863, 95% CI [0.832, 0.895], p < 0.001). Working status also remained protective, with employed individuals having significantly lower odds of DM (OR = 0.693, 95% CI [0.583, 0.824], p < 0.001). However, sex (OR = 0.918, 95% CI [0.783, 1.077], p = 0.293), Hispanic ethnicity (OR = 0.369, 95% CI [0.131, 1.040], p = 0.059), immigrant status (OR = 1.070, 95% CI [0.754, 1.518], p = 0.705), and marital status (OR = 1.062, 95% CI [0.903, 1.249], p = 0.467) were not significantly associated with DM. Importantly, the interaction term between ethnicity and education was significant (OR = 1.126, 95% CI [1.021, 1.241], p = 0.017), indicating that the protective effect of education on DM was weaker for Latino individuals compared to non-Latinos. Additionally, Black individuals continued to exhibit higher odds of DM (OR = 1.715, 95% CI [1.344, 2.189], p < 0.001). These results suggest that while education generally reduces the risk of DM, the strength of this protective effect is diminished for Latino individuals, in line with the Minorities’ Diminished Returns (MDRs) framework.

## 4. Discussion

The purpose of this study was to investigate whether the relationship between educational attainment and diabetes mellitus (DM) varies between Latino and non-Latino individuals, using data from the Understanding America Study (UAS) [39, 85–88]. In line with the Minorities’ Diminished Returns (MDRs) framework, we hypothesized that although higher educational attainment would be associated with lower odds of DM for both groups, the protective effect would be weaker for Latinos. The MDRs theory posits that the positive impact of socioeconomic resources, like education, is less substantial for marginalized groups when compared to non-minority populations.

The results confirmed our hypothesis. While increased educational attainment was associated with lower odds of DM for both Latino and non-Latino individuals, the health advantages of education—specifically the reduction in DM prevalence—were smaller for Latinos. This finding is consistent with previous research on other marginalized populations, such as Black Americans [32–36], and supports the MDRs framework by suggesting that structural factors may limit the health benefits of education for Latinos [89]. For example, a recent study using data from the 2022 National Health Interview Survey (NHIS) examined how educational attainment impacts marital status, employment, and food insecurity among Latino and non-Latino adults. This study found that education’s protective effects against food insecurity were weaker for Latinos, partially due to lower marriage and employment rates. These results emphasize the structural barriers that prevent Latinos from fully benefiting from education, not only in terms of health but also in socioeconomic outcomes [89].

A wealth of research supports the idea that education plays a crucial role in promoting cardiometabolic health outcomes [18, 29, 30]. Higher education is linked to better health literacy, improved diet, and greater access to health-promoting resources, all of which contribute to better cardiometabolic health [18, 29, 30]. Education also helps individuals manage stress and develop problem-solving skills, enabling them to navigate health challenges more effectively. Many studies have documented that each additional year of schooling is associated with improved cardiometabolic health outcomes, underscoring the importance of education in promoting physical well-being across the lifespan [18, 29, 30].

Research on MDRs has consistently shown that the benefits of education and other socioeconomic resources are less pronounced for ethnic minorities, especially Black individuals [37, 41, 44, 47, 51, 56, 90–95]. Although higher education generally leads to improved outcomes—such as higher income, better mental health, and enhanced physical health—the benefits are often smaller for Black individuals compared to White people [45, 72, 96, 97]. For instance, Black individuals with higher education frequently earn less and hold lower-quality jobs than their White peers, reducing the health benefits associated with education [73, 98, 99]. While much of the existing research on MDRs has focused on Black populations, limited attention has been given to Latinos, particularly concerning the effects of education on health outcomes like DM.

Research on how MDRs apply to Latino populations, especially regarding DM, remains sparse. While some studies have acknowledged that Latinos face similar structural barriers as other marginalized groups—such as discrimination and limited economic opportunities—the specific impact of these challenges on the health benefits of education is underexplored [100–103]. This study contributes to the growing body of MDRs research by providing evidence that the health benefits of education, particularly related to cardiometabolic health outcomes, are diminished for Latinos. This highlights the need for further research on how MDRs affect Latinos, especially in relation to physical health.

Several factors could explain why Latinos experience reduced health benefits from education. Structural inequalities likely play a major role, as systemic barriers limit access to high-quality education, healthcare, and job opportunities, even for those with higher educational attainment [104–106]. Social marginalization, economic instability, and discrimination may also contribute to increased chronic stress among Latinos, negatively affecting their physical health over time [107–109]. Additionally, labor market discrimination may hinder Latinos from securing jobs that promote a healthy lifestyle, further weakening the positive health effects of education [110–112]. Poor nutrition, higher rates of food insecurity, and reduced access to health-promoting resources further exacerbate these challenges, creating an environment where the protective effects of education on cardiometabolic health, such as DM, are diminished for Latinos [113, 114].

### 4.1. Implications

The findings from this study have important implications for both public health and educational policy. Improving physical health outcomes among Latino populations requires more than just increasing educational access; policymakers must also address the structural barriers, such as labor market discrimination and limited job opportunities, that prevent Latinos from fully benefiting from their educational achievements. Additionally, public health initiatives aimed at improving nutrition, access to healthcare, and social support systems may help reduce the impact of chronic stress and economic insecurity on Latino health outcomes. Tailored interventions addressing these structural and social determinants of health are essential for improving cardiometabolic outcomes in Latino populations.

### 4.2. Limitations

This study has several limitations. First, the sample was limited to English-speaking UAS participants, which may not fully capture the diversity of the broader U.S. Latino population, particularly those with lower levels of acculturation or non-English speakers. Second, while the analysis controlled for various demographic factors, other potential confounders, such as early-life socioeconomic conditions or childhood education access, were not included. Finally, the cross-sectional design of the study limits the ability to draw causal conclusions about the relationship between education and DM. Longitudinal studies are needed to confirm these findings and explore how the association between education and DM evolves over time.

## 5. Conclusion

This study provides new evidence supporting the theory of Minorities’ Diminished Returns (MDRs), demonstrating that the health benefits of education, particularly in reducing DM prevalence, are less pronounced for Latino individuals compared to non-Latinos. While education remains an important determinant of health, its benefits are not equally distributed across ethnic groups. Structural inequalities, chronic stress, and adverse socioeconomic conditions likely contribute to the reduced health returns of education for Latinos. Addressing these disparities requires multi-level interventions that target both individual and systemic factors to promote health equity and improve outcomes for marginalized populations.

## Figures and Tables

**Table 1.** Descriptive data

	All (n = 6782)		Non-Latino (n = 5952)		Latino (n = 830)	
	Mean	Std. Deviation	Mean	Std. Deviation	Mean	Std. Deviation
Age (Yrs) *	48	16	50	16	39	14
Education Years*	11.10	2.270	11.21	2.222	10.34	2.459
	n	%	n	%	n	%
Ethnicity						
Non-Latino	5,953	87.8	5,953	100	0	0
Latino	830	12.2	0	0	830	100
Race						
White	6,141	90.5	5,336	89.6	805	97.0
Black	642	9.5	617	10.4	25	3.0
Gender*						
Men	2,886	42.5	2,606	43.8	280	33.7
Women	3,897	57.5	3,347	56.2	550	66.3
Married*						
No	2,904	42.8	2,495	41.9	409	49.3
Yes	3,877	57.2	3,456	58.1	421	50.7
In Labor market*						
No	2,833	41.8	2,511	42.2	322	38.8
Yes	3,945	58.2	3,437	57.7	508	61.2
US Born						
No	435	6.4	217	3.6	218	26.3
Yes	6,348	93.6	5,736	96.4	612	73.7
Diabetes						
No	6,003	88.5	5,254	88.3	749	90.2
Yes	780	11.5	699	11.7	81	9.8

* p<0.05 for comparison of Latino and non-Latino

**Table 2.** Summary of regression without the interaction

	OR	95% CI		P
Age	1.038	1.032	1.044	< .001
Sex (Female)	.922	.787	1.081	.320
Years (Education)	.877	.848	.907	< .001
Hispanic	1.217	.922	1.607	.166
Immigrant	1.134	.798	1.610	.484
Working	.696	.585	.826	< .001
Married	1.066	.907	1.253	.440
Black	1.719	1.347	2.193	< .001
Intercept	.084			< .001

**Table 3.** Regression Results with Interaction Term

	OR	95% CI		p
Age	1.038	1.032	1.044	< .001
Sex (Female)	.918	.783	1.077	.293
Years (Education)	.863	.832	.895	< .001
Hispanic	.369	.131	1.040	.059
Immigrant	1.070	.754	1.518	.705
Working	.693	.583	.824	< .001
Married	1.062	.903	1.249	.467
Black	1.715	1.344	2.189	< .001
Ethnicity × Education	1.126	1.021	1.241	.017
Intercept	.105			< .001
